# Clinical Utility of Follow-Up Blood Cultures Among Adult Cancer Patients with Gram-Negative Bacilli Bacteremia

**DOI:** 10.1017/ash.2024.224

**Published:** 2024-09-16

**Authors:** Wesley Tang, Nancy Vuong, Ying Jiang, Natalie Dailey Garnes

**Affiliations:** Baylor College of Medicine; The University of Texas MD Anderson Cancer Center

## Abstract

**Background:** Gram-negative bacilli (GNB) bacteremia is a common and potentially fatal infection with mortality rates estimated to be 14-34%, despite effective antimicrobial treatments. Follow-up blood cultures (FUBCs) are blood cultures that are repeated after an initial positive culture and are indicated in certain clinical scenarios, such as in candidemia or Staphylococcus aureus bacteremia to ensure clearance of the bloodstream infection. FUBCs are used in bacteremia to assess the appropriateness and duration of antimicrobial therapy. Currently, there are no guidelines in place regarding the use of FUBC for GNB bacteremia. Furthermore, the utility of FUBCs is not well-studied in adults living with cancer. The purpose of this study is to identify risk factors associated with persistent GNB bacteremia in adult patients with cancer. **Methods:** We conducted a single-center, retrospective study in patients aged ≥18 years, hospitalized during calendar year 2022, living with cancer, and with laboratory confirmed GNB bloodstream infection. FUBC was defined as a blood culture performed within 7 days of the initial positive blood culture. Patients were grouped as having the same organism on FUBC (+/same), FUBC with no growth (NG) (+/NG), and different organism on FUBC (+/Different). Patients with a different organism on FUBC were excluded from analysis. We gathered demographic information, suspected source of bacteremia, type of malignancy, identified organisms, presence of antimicrobial resistance, and comorbidities (eg. presence of central venous catheters, urinary catheters, end-stage renal disease). Categorical variables were compared using Chi-square or Fisher’s exact test. Continuous variables were compared using Wilcoxon rank sum tests. Logistic regression analysis was used to identify the independent predictors of persistent GNB bacteremia. **Results:** 356 unique patients with FUBC were identified after inclusion/exclusion criteria. 93/356 (26%) of patients had persistent GNB bacteremia (+/same). Multivariate analysis identified history of bacteremia within the preceding year (OR 2.95, 95% CI [1.6-5.6]) and Achromobacter spp. bacteremia (OR 10.03, 95% CI [1.59-63.23] as independent risk factors for persistence. Organisms with multidrug resistance such as extended-spectrum beta-lactamase (OR 2.47, 95% CI [1.21-5.07]) and carbapenem-resistant organisms (OR 3.35, 95% CI [1.04-10.81]) were also associated with persistent GNB bacteremia. **Conclusions:** This is the first study to specifically identify risk factors for persistent GNB bacteremia in patients living with cancer. FUBC may be useful in GNB bacteremia with less common organisms and/or if they exhibit multidrug resistance on susceptibility testing. The utility of FUBC should be further explored in patients with cancer with certain risk factors.

